# MicroRNA profiling identifies a novel compound with antidepressant properties

**DOI:** 10.1371/journal.pone.0221163

**Published:** 2019-08-23

**Authors:** Stacy L. Sell, Deborah R. Boone, Harris A. Weisz, Cesar Cardenas, Hannah E. Willey, Ian J. Bolding, Maria-Adelaide Micci, Michael T. Falduto, Karen E. O. Torres, Douglas S. DeWitt, Donald S. Prough, Helen L. Hellmich

**Affiliations:** 1 Department of Anesthesiology, University of Texas Medical Branch, Galveston, Texas, United States of America; 2 Graduate School of Biomedical Science, University of Texas Medical Branch, Galveston, Texas, United States of America; 3 University of Mississippi Medical Center: Psychiatry & Human Behavior, Jackson, Mississippi, United States of America; 4 GenUs BioSystems, Northbrook, Illinois, United States of America; 5 Paradise Genomics, Inc., Northbrook, Illinois, United States of America; University of Florida, UNITED STATES

## Abstract

Patients with traumatic brain injury (TBI) are frequently diagnosed with depression. Together, these two leading causes of death and disability significantly contribute to the global burden of healthcare costs. However, there are no drug treatments for TBI and antidepressants are considered off-label for depression in patients with TBI. In molecular profiling studies of rat hippocampus after experimental TBI, we found that TBI altered the expression of a subset of small, non-coding, microRNAs (miRNAs). One known neuroprotective compound (17β-estradiol, E2), and two experimental neuroprotective compounds (JM6 and PMI-006), reversed the effects of TBI on miRNAs. Subsequent *in silico* analyses revealed that the injury-altered miRNAs were predicted to regulate genes involved in depression. Thus, we hypothesized that drug-induced miRNA profiles can be used to identify compounds with antidepressant properties. To confirm this hypothesis, we examined miRNA expression in hippocampi of injured rats treated with one of three known antidepressants (imipramine, fluoxetine and sertraline). Bioinformatic analyses revealed that TBI, potentially via its effects on multiple regulatory miRNAs, dysregulated transcriptional networks involved in neuroplasticity, neurogenesis, and circadian rhythms- networks known to adversely affect mood, cognition and memory. As did E2, JM6, and PMI-006, all three antidepressants reversed the effects of TBI on multiple injury-altered miRNAs. Furthermore, JM6 reduced TBI-induced inflammation in the hippocampus and depression-like behavior in the forced swim test; these are both properties of classic antidepressant drugs. Our results support the hypothesis that miRNA expression signatures can identify neuroprotective and antidepressant properties of novel compounds and that there is substantial overlap between neuroprotection and antidepressant properties.

## Introduction

Major depressive disorder (MDD) is the most common psychiatric disorder encountered after a traumatic brain injury (TBI) [1]. Prevalence rates for depression after TBI are from 20–80% with the overall average at 31% compared to 8–10% for the general population [2]. This increase in prevalence starts early after injury, remains elevated for an extended period, and does not necessarily correlate with severity of injury. The presence of depression-like symptoms is associated with worse outcomes in the first six months [3], and out to seven years after injury [4]. Impaired psychosocial function is documented from 1–3 years after even a mild TBI [5], including increased aggression and suicidal thoughts [6, 7].

Although more than five million survivors of TBI live with chronic disability, and co-morbid depression undermines rehabilitation efforts, little evidence is available to guide treatment of depression after TBI; studies are few with mixed results [8]. Antidepressants are considered off-label for depression in TBI patients [9] and some are associated with adverse effects; for instance, tricyclic antidepressants (TCAs) increase the risk of seizures [10]. However, there are some encouraging reports. Two studies of patients with TBI and depression demonstrated clinical effectiveness using desipramine, a TCA [11], and sertraline, a selective serotonin-reuptake inhibitor (SSRI) [12]. Nonetheless, the paucity of treatments for TBI and/or depression attests that research to develop new treatments should be a high priority.

In genome-wide expression profiling studies, we found that TBI altered the expression of small, noncoding microRNAs (miRNAs) that are associated with genes related to neuronal homeostasis and psychiatric disorders such as depression [13, 14]. Since miRNAs can inhibit gene translation by binding to 7-base-pair seed regions, a single miRNA can bind to and regulate the expression of hundreds of genes which contain that binding site, suggesting that the translation of hundreds of genes can be modulated by a single miRNA [15]. Many of these miRNAs act as master molecular switches to turn on or off entire genetic programs [16, 17]. Studies showing that TBI alters miRNA levels in humans [18] and that miRNAs such as miR-134 can regulate the size of dendritic spines and potentially, synaptic plasticity [19], suggest that recovery of function after TBI may be facilitated by therapeutic modulation of miRNAs.

MicroRNAs and their associated gene targets are dysregulated in Huntington’s disease (HD), [20, 21]. In a transgenic mouse model of HD, a novel compound, 2-(3,4-dimethoxybenzenesulfonylamino)-4-(3-nitrophenyl)-5-(piperidin-1-yl)methylthiazole (JM6), prevented synaptic loss and behavioral deficits by decreasing microglial activation (neuroinflammation) and glutamate levels while increasing levels of kynurenic acid (KYNA) in the brain [22]. Because inflammation is a hallmark of neurodegenerative diseases [23], our interest in JM6 originally stemmed from its anti-inflammatory properties. However, dysfunction of the kynurenine pathway is implicated in major depression [24] and KYNA was shown to be neuroprotective after TBI in rats [25]. Thus, JM6 appeared to possess anti-inflammatory, neuroprotective and antidepressant properties, making it an ideal therapeutic candidate.

**Fig 1:**
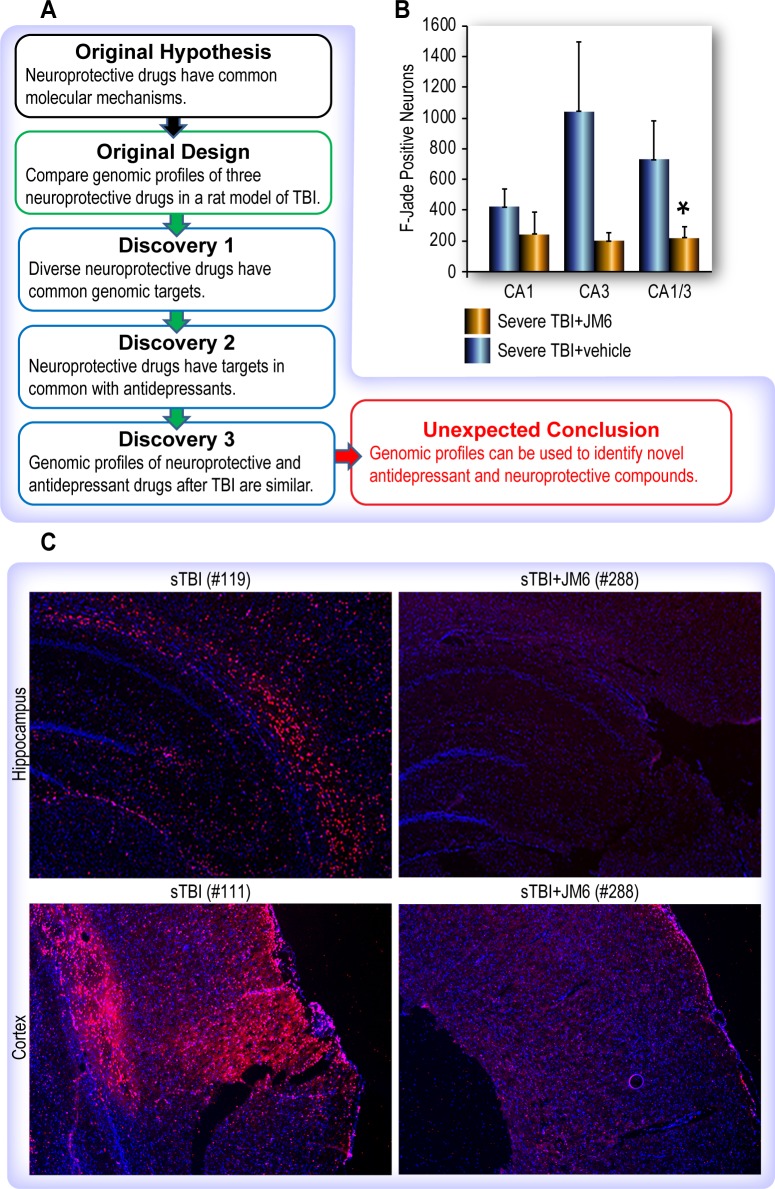
Schematic of experimental workflow and pilot studies. (A) The original hypothesis that diverse drugs with neuroprotective properties share common molecular mechanisms was validated by analysis of genome-wide microRNA expression profiles induced by three different drugs in rat brains after TBI. Unexpectedly, we found these microRNA profiles to be similar to those induced by antidepressant drugs. (B) Stereological assessment of neuronal injury after JM6 treatment reveals neuroprotective effect of JM6. JM6 treated rats had reduced numbers of FJC+ neurons in the CA1 (p = 0.19) and CA3 (p = 0.07) sub-regions of the hippocampus. The overall average (CA1 and CA3 taken together) was significantly different *p = .038 vs untreated. (C) Immunohistochemistry using CD11b (OX-42) shows JM6 reduced microglial activation after TBI. Increased microglial activation after TBI (left) is ameliorated by treatment with JM6 (right) in the rat hippocampus (upper) and cortex (lower). Note, in sham-injured brains (not shown), staining was undetectable.

Here, we initially set out to test the hypothesis that diverse neuroprotective compounds act on a common set of molecular targets. We found that JM6, 17β-estradiol (E2), and PMI-006 produced similar hippocampal miRNA expression profiles after injury, and *in silico* analysis suggested that the miRNAs altered by these drugs regulate genes involved in depression. Thus, we further hypothesized that these drug-induced miRNA expression profiles predicted antidepressant effects. We tested this hypothesis by investigating the post-TBI hippocampal miRNA expression profile of three clinically used antidepressants, and testing JM6 in the forced swim test. Our results suggest that novel drug compounds with neuroprotective and/or antidepressant properties can be identified by their miRNA expression profiles (Fig 1A).

## Methods

### Animals

Adult male Sprague-Dawley rats (300–400 g) were obtained from Charles River Laboratories (Wilmington, MA), housed two per cage with food and water ad libitum, and maintained at a constant temperature (21^o^ – 23°C) and humidity (40–60%) with lights on 06:00–18:00. Rats were anesthetized with 4% isoflurane and prepared for parasagittal fluid-percussion TBI as previously described [26]. All experiments were conducted in accordance with the National Institutes of Health’s *Guide for the Care and Use of Laboratory Animals*, *Eighth Edition* (National Research Council, 2011) and approved by the Institutional Animal Care and Use Committee of the University of Texas Medical Branch, Galveston, Texas.

### Pilot study of neurodegeneration: Fluoro-Jade C staining and stereological counting

Rats (n = 3/group) received either JM6 (400mg/kg) or vehicle (sterile water, 1 ml) by oral gavage, one hour after injury. Twenty-four hours after injury, brains were removed and frozen on dry ice. Coronal sections (25 um) were cut on a cryostat with every fifth section collected on a plus slide. Sections were stained with 0.0001% Fluoro-Jade C, a marker of injured, degenerating neurons and counter stained with 1% cresyl violet. Fluoro-Jade-C positive (FJC+) neurons in the CA1-CA3 regions of the hippocampus were counted using Stereo-Investigator software (Version 9, MBF Bioscience, Williston, VT). Total number of injured neurons was calculated using the Optical Fractionator probe (MBF Bioscience). Parameters for counting FJC+ neurons were: intervals (x = 400; y = 400) with a counting frame (x = 400 μm; y = 400 μm) superimposed on the tissue section image. This procedure sampled 100% of all FJC+ neurons. An average number of 50 sections were counted in each treatment group. The total number of injured neurons estimated in each treatment group was compared to the total number of injured neurons estimated in each control group and an untreated TBI group.

### Pilot study of neuroinflammation: Immunohistochemistry for assessment of microglial activation

A marker of microglial activation, an antibody to CD11b (OX-42), was used to identify areas of inflammation in the rat brain. Three groups of rats (n = 3/group) were used to assess the effects of JM6 on TBI-induced inflammation in the hippocampus using immunohistochemistry: 1) TBI; 2) SHAM; 3) TBI + JM6. Rats were prepared and treated as above. Twenty-four hours after injury, rats were perfused with 4% paraformaldehyde, brains collected and 10-μm frozen sections were cut on a cryostat. Sections were then incubated overnight with a primary antibody (mouse anti-CD11b; 1:2000, BD Biosciences, San Jose, CA). The following morning, sections were rinsed and then incubated with a secondary antibody (Alexa 594 goat anti-mouse; 1:400, Life Technologies, Grand Island, NY) at ambient temperature, and then mounted with DAPI (marker of nuclei) for imaging. An Olympus BX51 Fluorescent Microscope was used to visualize the hippocampal formation and surrounding cortical regions.

### Microarray analysis of neuroprotective compounds after TBI

Rats (N = 18) were divided into six treatment groups (n = 3/group): 1) Naïve (no injury and no treatment; 2) SHAM (anesthetized and prepared for TBI but uninjured); 3) TBI (anesthetized and received FPI injury as described above); 4) TBI + JM6 (400 mg/kg); 5) TBI + PMI-006 (250 mg/kg); and TBI+ estradiol (33 ug/kg, IP). Twenty-four hours after injury or equivalent, rats were anesthetized again with isoflurane, brain tissue was collected and the hippocampus was isolated. Total RNA was extracted, purified from hippocampal tissue using Ribopure kit (Ambion), quantified by UV spectrophotometry (OD 260/280) and RNA quality was assessed using an Agilent Bioanalyzer, RNA integrity (RIN) values were greater than 9 for all samples. Gene expression (GE, Agilent Rat v3 GE 4x44K) and miRNA (Agilent miRNA 8x15K) arrays were performed as described in Rojo et al., [13] and Boone et al., [14]. Data was analyzed with Agilent Feature Extraction and GeneSpring GX v7.3.1 software packages (Agilent Technologies, Santa Clara, CA). To compare individual expression values across arrays, raw intensity data from each gene or miRNA was normalized to the 75th percentile intensity of probes above background on each array. Genes or miRNAs with values greater than background intensity in all replicates of at least one condition were filtered for further analysis. Differentially expressed genes or miRNAs were identified with T-test p-values < 0.05 and fold change > 1.2 fold. It is our standard practice to not report a false discovery rate (FDR) because any Multiple Testing Correction (MTC) algorithm will severely limit the number of probes that appear to be differentially expressed (creating false negatives). MTC upsets the balance between false positives and false negatives necessary in analyzing microarray data. MTC essentially adjusts the p-values while keeping the rank order of probes intact. Using the combination of quality probe filtering, fold change filtering, and a moderate p-value is a better balance of false positives and false negatives. In support of this, the US FDA sponsored MAQC study concluded that there is better reproducibility when genes are ranked on fold change with a non-stringent p-value cutoff rather than p-value alone (Shi L., et al., Nature Biotechnology, 24:9, p1151-1161, 2006, see page 1158 1st full paragraph). Gene and miRNA microarray data has been deposited in the National Center for Biotechnology Information Gene Expression Omnibus (Accession numbers: GSE59645, GSE59646, GSE115614).

### Bioinformatic analyses with Ingenuity Pathway Analysis and Qlucore Omics Explorer

Fold change ratios that had significant *P*-values for TBI+JM6 vs. TBI genes and miRNA transcripts from Agilent rat microarray results were imported into Ingenuity Pathway Analysis(IPA) software (QIAGEN Bioinformatics). In this comparison, 1,270 mRNA probes for TBI >2 fold vs sham and 33 miRNA probes for TBI >1.2 fold vs sham, mapped to the IPA database. Of these, 25 TBI and/or JM6 affected miRNAs regulate (i.e. potentially suppress expression of) 518 TBI-associated mRNAs. Top canonicals pathways in immune response, disease signaling, and nervous system signaling enriched in these 518 genes are reported. Qlucore Omics Explorer (QOE, Qlucore, Lund, Sweden), a bioinformatics software platform (based on R) that allows a dynamic and interactive visualization of multivariate data by projecting high dimensional data down to lower dimensions, was used for principal component analysis.

### Microarray analysis of antidepressant effects in TBI

Rats were surgically prepared, treated with an antidepressant or vehicle (N = 3/group), brain tissue collected and whole genome microarray analyses were performed as described above (see *Microarray analysis*). All three antidepressants (fluoxetine, imipramine, and sertraline) were given at a dose of 10 mg/kg, i.p., one hour after injury or sham-injury. Tissue was collected 24 h after injury or sham-injury.

### Forced swim test (FST)

The FST was validated, prior to testing the antidepressant efficacy of JM6, using the antidepressant imipramine (15 mg/kg IP). Male Sprague-Dawley rats (300–350 g) received either imipramine (n = 5) or vehicle (n = 6) 24, 5 and 1 h prior to the test swim. For the JM6 experiment, Rats received two weeks of daily oral gavage with vehicle (n = 9) or JM6 (100 or 200 mg/kg; n = 10) [22], prior to the test swim. The FST was conducted as described in Sell et al., (2008) [27]. Rats were first introduced to the swim cylinder for 15 min, and 24 h later, subjected to a video-recorded 5 min test session. Videos were scored by three treatment-blind observers to quantify swimming, climbing and immobility, as described by Lucki [28]; the predominant behavior was noted during each of 60 five second intervals. From these scores, the latency to adopting immobility posture was measured by counting the 5 second bins until the first bin in which immobility was recorded.

### Statistical analysis

Stereological counts of FJC+ neurons were analyzed using a Student’s t-test comparing two groups: JM6 treated vs. untreated. For IPA analyses, comparisons had to pass a t-test *P*-value cutoff of 0.05. Core analyses were conducted on the 518 genes that were differentially expressed in TBI+JM6 vs. TBI and targeted by a miRNA differentially expressed in the opposite direction in the same samples. For the FST, mean scores for each behavior were calculated and analyzed using a one-way analysis of variance (ANOVA).

## Results

### Pilot studies

To validate the neuroprotective effects of JM6 prior to molecular analysis, we tested it using the fluid-percussion model of brain injury in two pilot studies. First, we assessed neurodegeneration in the rat hippocampus after injury with and without JM6 treatment using stereological quantification of FJC+ neurons in the CA1/2 and CA3 sub-regions. Reduced numbers of dying neurons throughout the CA1/2 and CA3 regions of the hippocampus were observed in the JM6 treatment group (Fig 1B).

Second, we performed immunohistochemical analysis of TBI brains using CD11b (OX-42), a marker of microglial activation which is an indicator of neuroinflammation. Treatment with JM6 reduced microglial activation 24 hours after injury in both the hippocampus and cortex (Fig 1C). Taken together, evidence from these two pilot studies support the conclusions drawn from previous studies that JM6 has neuroprotective properties that are manifested through reduced neuronal injury and neuroinflammation.

### MicroRNA expression profiling and principal component analysis (PCA) of rat hippocampus reveals similar expression profiles in neuroprotective drug-treated rats

**Fig 2:**
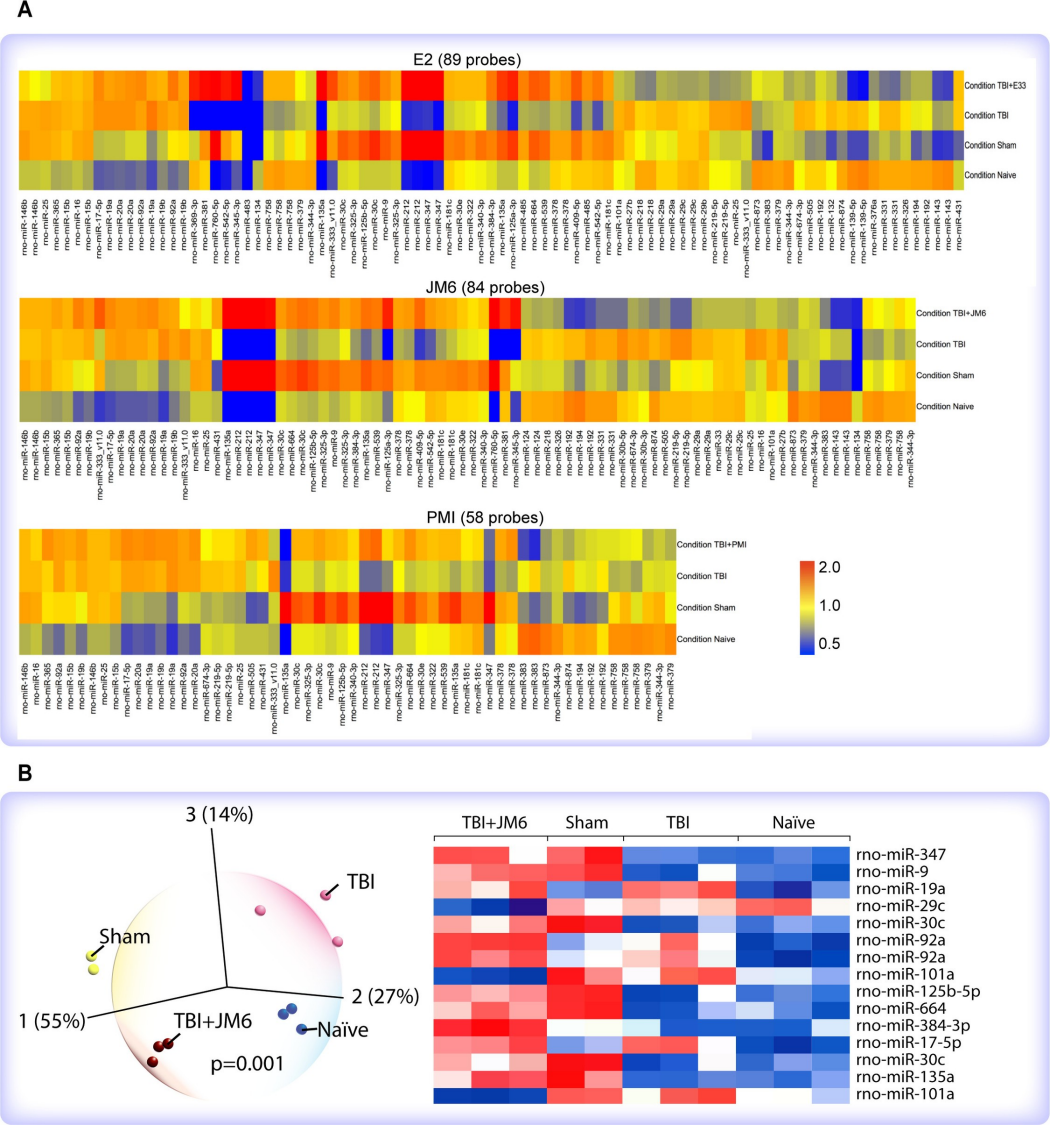
Agilent microRNA microarray analysis. (A) Heatmaps of genome-wide miRNA profiles of control and TBI rats with and without treatment with either E2 (upper), JM6 (middle) or PMI-006 (bottom), demonstrated that after TBI, each drug restored miRNA expression profiles to patterns that are similar to sham-injured (no TBI) rats. (B) Dynamic principal component analysis (PCA; left) and hierarchical clustering (right) in Qlucore Omics Explorer showing that injured rats treated with JM6 exhibit miRNA expression patterns that resemble those of sham-injured rats. PCA shows that Naïve, Sham, TBI and TBI+JM6 are all distinguishable one from another.

Previously, we demonstrated that PMI-006 decreased FJC+ staining and reduced microglial activation in rats after TBI [29] and the neuroprotective properties of E2 are established (for review, see Engler-Chiurazzi et al., 2016 [30]). MicroRNA microarray profiling of naïve, sham-injured, TBI or TBI plus drug treatments (JM6, E2 or PMI) revealed that all three neuroprotective drugs restored TBI induced miRNA profiles to sham-injury patterns (Fig 2A). For instance, the heatmaps show that all three drugs restored sham-control expression levels of miR-212.

Qlucore Omics Explorer 3.4 (Qlucore AB, Lund, Sweden) uses dynamic principal component analysis (PCA) based on a powerful statistical platform (R) to allow visualization of multidimensional data and test hypotheses about significant differences among experimental groups. Principal component analysis confirmed our hypothesis that injured rats treated with JM6 (TBI+JM6), were more similar to naïve and sham-injured rats than to injured rats (TBI) (Fig 2B, left). Hierarchical clustering shows the TBI+JM6 group to be more similar to Sham than to TBI (Fig 2B, right).

### JM6 affects pathways linked to neurogenesis/neurodegeneration and depression

**Fig 3:**
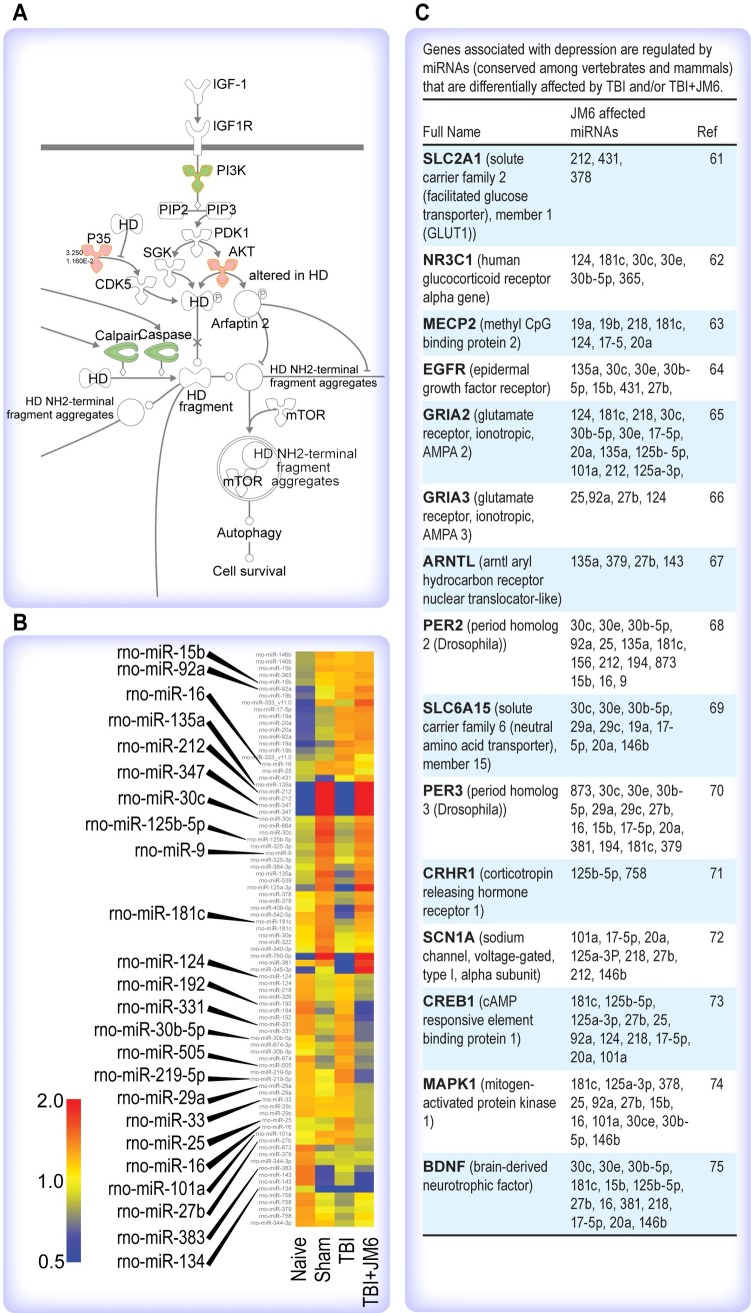
Ingenuity pathway analysis (IPA) of JM6 effects on Huntington’s disease (HD) pathway. (A) JM6 treatment downregulates the expression of HD genes associated with cell death, calpain and caspase (green) and upregulates a gene associated with cell survival, *Akt* (red). (B) In silico analysis of drug-affected miRNAs after JM6 treatment, miRNAs affected by TBI and returned to sham patterns by JM6 treatment (shown enlarged) are associated with depression-linked genes. (C) List of genes associated with depression are regulated by broadly conserved miRNAs affected by TBI and/or TBI+ JM6.

Since JM6 was originally investigated as a potential therapy for HD, we chose the HD pathway for analysis (Fig 3A). In hippocampi of TBI rats, JM6 treatment downregulated the expression of calpain and caspase, two genes involved in apoptotic cell death [31, 32] and upregulated *Akt*, a pro-survival gene [33].

*In silico* analysis of differentially expressed miRNAs after drug treatments revealed a large number of differentially expressed miRNAs in TBI animals, both treated and untreated after injury, that have previously been linked to depression (Fig 3B). A closer examination of broadly conserved miRNA target genes that are predicted to be regulated by JM6 (Fig 3C) shows that many are expressed in limbic brain regions (e.g. amygdala and hippocampus) associated with depression. In particular, changes in brain expression of *Creb1* and *Bdnf* are closely linked to depression pathology and antidepressant efficacy [34, 35]. For instance, ketamine can rapidly act as an antidepressant and works, in part, by increasing the synaptic release of BDNF in human patients [36].

**Fig 4:**
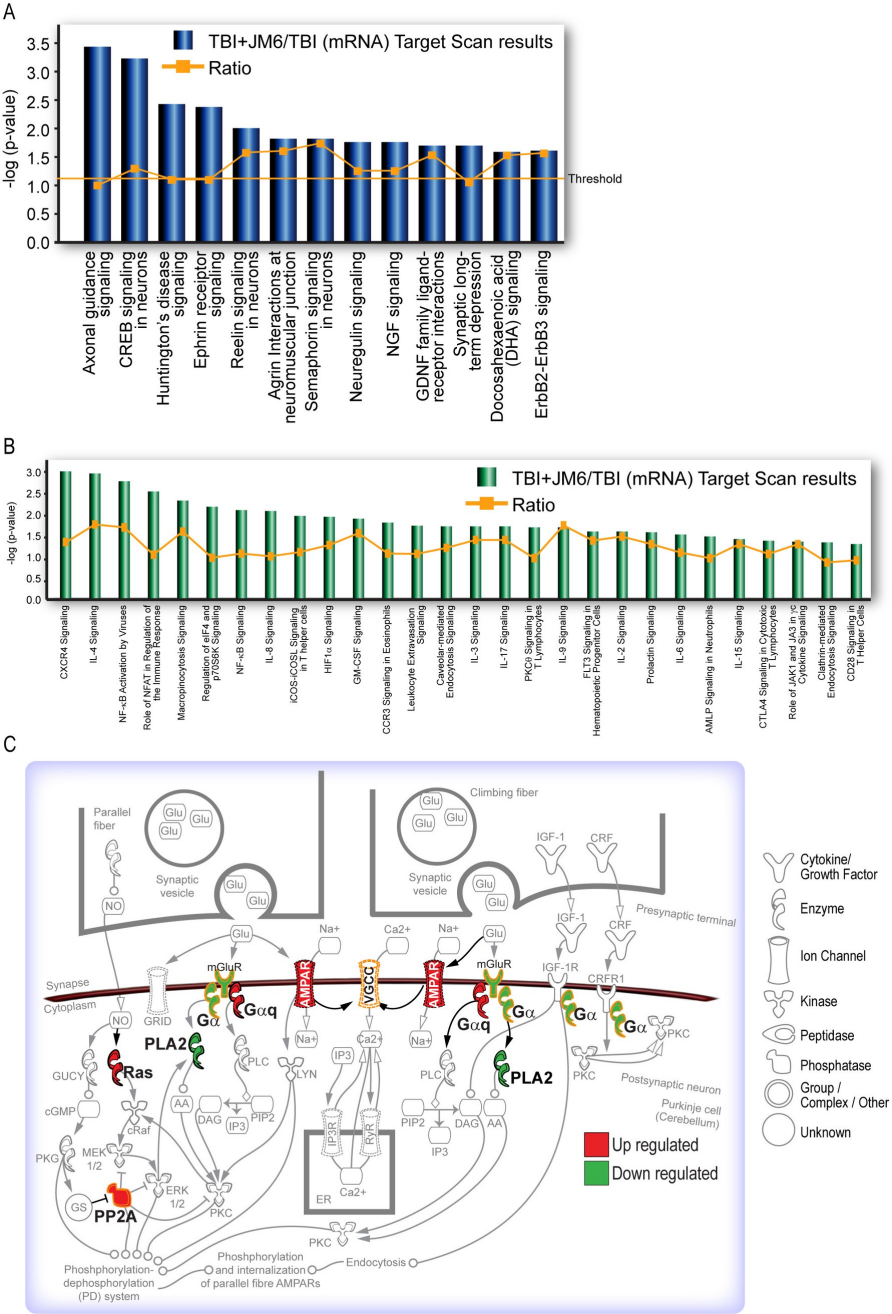
Ingenuity pathway analysis after JM6 treatment. (A) JM6 treatment significantly affects genes in pathways linked to depression, including axonal guidance, CREB and Huntington’s disease signaling. (B) The top immune response pathways affected by JM6 treatment are also linked to depression, including CXCR, IL-4 and NF-κB activation by virus signaling. Threshold indicates minimum significance level [–log (p-value) from Fisher’s exact test]. Ratio refers to the number of molecules from the dataset that map to the pathway listed, divided by the total number of molecules that define the canonical pathway from within the IPA knowledgebase. (C) JM6 influences the synaptic long-term depression pathway by upregulating AMPA receptor signaling.

Correlating the miRNA and mRNA expression data using IPA’s miRNA target filter, we found the top disease-associated pathways affected by JM6 treatment (e.g. axon guidance, Creb signaling, ephrin receptor signaling, etc) are associated with depression in animal models and/or in human patients [37–39] (Fig 4A). Furthermore, drug treatment altered multiple immune response pathways that are also implicated in depression (Fig 4B, genes in significant canonical pathways shown in Supporting Information, S1 Table). Ingenuity pathway analysis of canonical cell signaling pathways indicated that JM6 treatment increases expression of AMPA receptors in the synaptic long-term depression pathway (Fig 4C). This supports the notion of antidepressant efficacy of JM6 since potentiation of AMPA receptor signaling is a central mechanism of rapid-acting antidepressants [40, 41].

### Three FDA approved antidepressants produce similar miRNA profiles to JM6 after TBI

**Fig 5:**
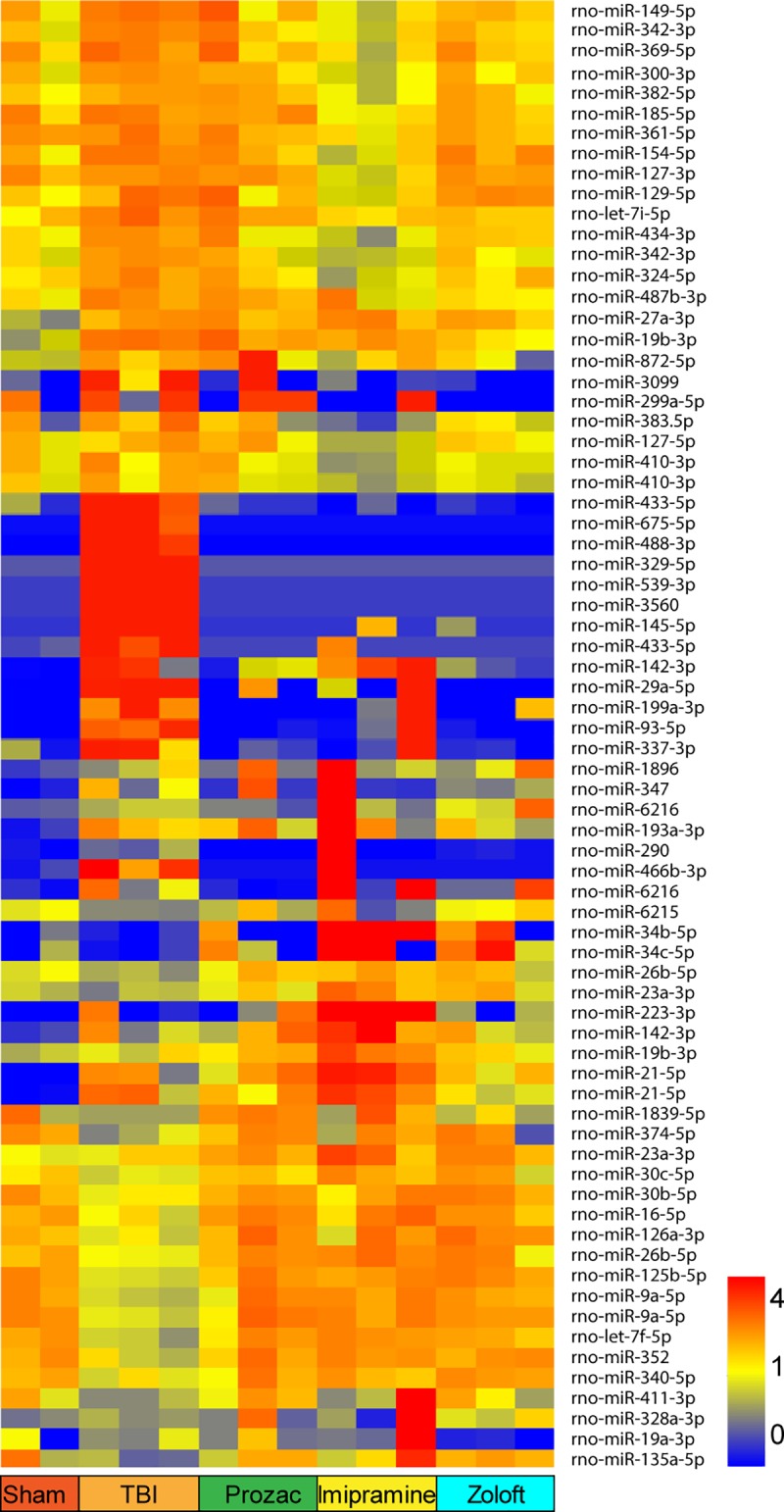
Heatmap showing hippocampal miRNA expression profiles of antidepressant-treated injured rats. Prozac (fluoxetine), imipramine and Zoloft (sertraline) restored TBI-induced miRNA expression profiles to those of sham-injured control rats. Probes differentially expressed in at least one of seven comparisons are displayed as normalized to the median expression across all 14 samples (72 probes, >1.2Fold, p<0.05).

**Fig 6:**
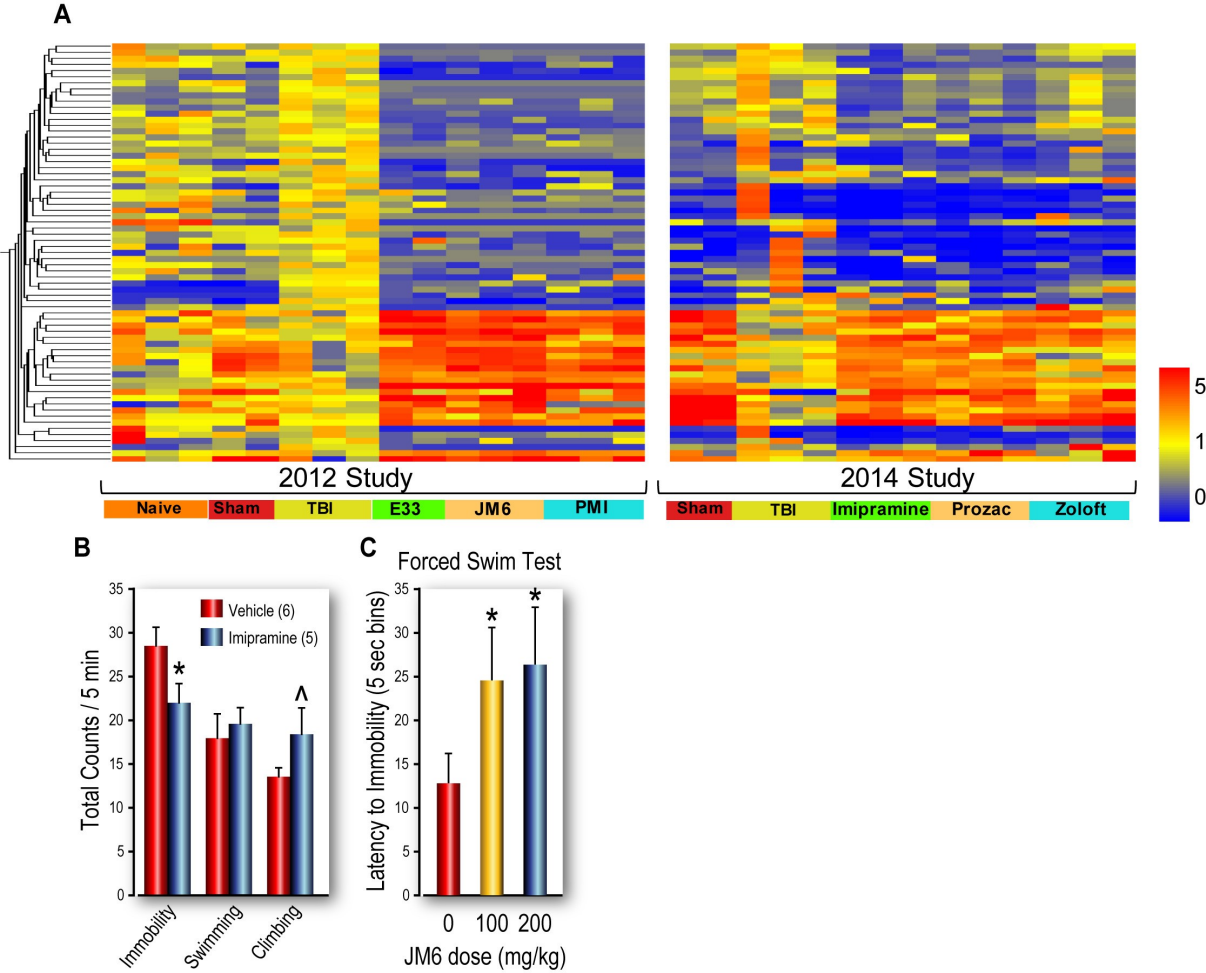
Heatmaps of drug-induced gene expression (GE), performed two years apart. (A) Hippocampal GE profiles from injured rats treated with fluoxetine, imipramine or sertraline, [data from 2014 (*right*), is from the same rats as in Fig 5] are similar to hippocampal GE profiles of rats treated with JM6, E2 or PMI, data from 2012 (*left*). Genes > 1.5 fold in the same direction in at least five drug treatments vs TBI and *p*-value < .05 in at least one drug treatment vs. TBI are displayed as normalized to the mean expression of the study specific TBI samples (69 probes). (B) The FST was used to test the antidepressant efficacy of JM6. First imipramine was tested to validate the depression assay. Imipramine (15 mg/kg) decreased immobility and increased climbing, in the forced swim test (FST). (C) While there were no significant effects on duration of climbing, swimming or immobility, JM6 had dose-dependent antidepressant effects on latency to immobility, The FST validates antidepressant efficacy of JM6 *p < .05.

Agilent miRNA and gene expression analysis of the hippocampus 24 h after injury plus antidepressant treatment demonstrated that treatment with all three antidepressants resulted in similar expression profiles (Figs 5 and 6B). Furthermore, as was found in TBI rats treated with neuroprotective drugs, the miRNA signatures obtained from antidepressant-treated injured rats resembled those of sham-injured rats (Fig 5). Although the initial gene expression studies with JM6, E2 and PMI-006 were performed two years prior (2012) to the studies with the three antidepressants (2014), heatmaps of gene expression for all six drug-treated groups are remarkably concordant (Fig 6A and 6B), suggesting that JM6, E2, PMI-006 and the three antidepressants act on a common set of gene targets.

The functional antidepressant effects of JM6 were assessed using the FST. First we validated the FST in our hands using imipramine (Fig 6B). Imipramine treatment reduced immobility and increased climbing, confirming antidepressant efficacy. JM6 treatment (100 and 200 mg/kg) significantly increased the latency to immobility, confirming its efficacy as an antidepressant (Fig 6C).

Taken together, our results support the following three ideas: 1) Drug compounds that reverse TBI-induced miRNA profiles and restore them to sham-injury-like profiles are potential candidates for neuroprotection. 2) Antidepressants show gene and miRNA expression profiles that indicate neuroprotective effects after TBI. 3) Comparison of miRNA profiles of experimental drug compounds with classical antidepressants provides a novel way to identify new antidepressant candidates.

## Discussion

Two key findings of our study are: one, drug compounds with neuroprotective properties act on common sets of molecular targets and two, the miRNA profile of a novel compound (JM6) predicted it would have antidepressant effects. The fact that JM6 and two neuroprotective compounds (PMI-006 and E2) induced similar genomic responses in hippocampal tissue after TBI suggests that molecular profiles could be useful for screening potential drug effects.

The finding that JM6 down-regulated the expression of calpain and caspase, two genes involved in apoptosis and neurodegeneration [42], and upregulated *Akt*, a gene involved in cell-survival [33], is further evidence that JM6 is neuroprotective. Taken together with the *in silico* analyses neuroprotective drug-induced miRNAs pointed to an association with depression; these findings have major implications for antidepressant drug discovery.

Target genes of the miRNAs that were altered by JM6 (e.g., *Creb*, *Bdnf* in Fig 3C) were found to be expressed in limbic brain areas (e.g. hippocampus) in previous studies [43, 44], and involved in the pathophysiology of depression and the response to antidepressants. A common set of genes involved in cell survival, synaptic plasticity and immune response appear to be modulated by both neuroprotective and antidepressant drugs, supporting the notion that neuroprotective and antidepressant responses are related and share common mechanisms.

This combined efficacy is not surprising in light of the neurodegeneration hypothesis of depression [45]. For many years the monoamine hypothesis of the pathophysiology of depression was the reigning explanation for the physiology of depression. The efficacy of monoaminergic compounds such as SSRIs further supported this hypothesis. However, despite the success of a variety of monoamine based therapies, complications remained that could not be explained by an imbalance in monoaminergic neurotransmission. For instance, the length of time it takes (2–3 weeks) for monoamine-based antidepressant treatment to be effective is incongruent with the pharmacokinetics of SSRIs, or other common antidepressants. Additionally, evidence suggested that depression involved more than just imbalances of monoamines. For example, the NMDA antagonist, ketamine showed antidepressant effects that appeared to act through enhancing synaptic plasticity [46], suggesting a role for other neurotransmitter systems such as GABA and glutamate. Indeed, a recent study of ketamine metabolites showed that the rapid antidepressant effects of these metabolites were mediated by an early and sustained activation of AMPA receptors [40]. This suggests that our finding that JM6-increased gene expression of AMPA receptors may partly explain JM6’s antidepressant effects.

Moreover, growing evidence for a role of stress in the onset of depression, such as stress-mediated hippocampal atrophy in human patients with depression [47] indicates a role for stress-induced neurodegeneration in depression. Thus, the neurodegeneration hypothesis of depression has taken center stage as the leading pathophysiological explanation for MDD. This idea is corroborated by the role of excitotoxic damage (via NMDA, Ca++, cAMP), impaired neurogenesis, and reduced levels of neurotrophins (CREB, BDNF, VEGF) in the hippocampus during depression [48]. Substantial evidence exists that antidepressants enhance neuroplasticity and neurogenesis. For example, treatment with fluoxetine, in rats, increased the number of pyramidal cell dendritic spine densities in the hippocampus [49]. And, chronic treatment with various types of antidepressants increases neurogenesis (e.g. SSRIs, SNRIs, MAOIs). Neurogenesis is required for the behavioral response to antidepressants [50] and increased neurogenesis in the hippocampus requires 14–21 days of treatment as well as activation of the cAMP-CREB cascade, possibly explaining the latency in efficacy of traditional antidepressants. Activation of CREB promotes neurogenesis through increasing levels of VEGF for proliferation and BDNF for survival of neural stem cells. The miRNAs affected by JM6 and antidepressants in our study (e.g. miR-212/132) have been implicated in regulating the gene expression of these neurotrophins as well as depression-associated genes such as the serotonin transporter (SERT) [51, 52].

Dysregulation of miRNAs is associated with depression [43, 53], hence miRNAs may be useful therapeutic targets and are indeed targets of effective antidepressants; the SSRI, fluoxetine decreases levels of miR-16 (regulates BDNF) in mouse hippocampus [54]. Since hippocampal miRNAs are a common target of varied antidepressant treatments such as acute ketamine, chronic fluoxetine and repeated electrical stimulation (EST) and their effects may be mediated through the BDNF-TrkB pathway, it is notable that genes in this pathway are regulated by multiple miRNAs in our study. However, antidepressant-induced effects on miRNA expression may only be evident following an external stressor, such as TBI or chronic mild stress [55]. Neuronal specific miRNAs (miR-124a, miR-132) are dysregulated in HD [21]. Our data showing that these two miRNAs are influenced by JM6 in injured rats suggests another mechanism for its neuroprotective effects; miR-124 regulates Sox 9 [56] and reduced expression of Sox 9 is necessary for differentiation of neural stem cells. Another miRNA involved in regulation of neural stem cells, miR-9, regulates the nuclear receptor 2E (NR2E) which has a role in regeneration and proliferation [57]. Additionally, miR-16 regulates SERT expression and has a role in the response to fluoxetine [58]. All of these miRNAs, that are neuronal specific and regulate genes involved in regeneration, proliferation and differentiation as well as SERT expression, are involved in the pathophysiology of MDD.

Some neuronal miRNAs that are dysregulated in MDD are located in the synapto-dendritic compartment. Turnover of these neuronal miRNAs is faster than in other cell types, allowing rapid adaptation to neuronal activity that may involve calpain, one of the genes altered by JM6. miR-124a, one of the most abundant miRNAs in the brain, regulates glucocorticoid receptors in the hippocampus and has a role in permitting neurogenesis to take place. miR-132 is upregulated in the dentate gyrus of the hippocampus in response to neuronal activity. Reciprocal antagonism between miRNAs and their target genes is a mechanism for regulating neural plasticity and regeneration (e.g. miR-124a/CREB, miR-132/BDNF, miR-134/BDNF). Taken together, these concepts provide evidence of a regulatory role of miRNAs in the pathophysiology of neurodegeneration after TBI, or other stressors, that after chronic exposure may lead to depression. These regulatory miRNAs may be useful therapeutic targets to reverse this injury- or stress-induced neurodegeneration through enhancing neuronal plasticity and neurogenesis.

Although JM6 does not cross the blood brain barrier (BBB), evidence suggests that peripheral inhibition of Kynurenine-3-monooxygenase (KMO) provides neuroprotection through accumulation of kynurenine in the blood. Kynurenine in the blood is actively transported through the BBB by a neutral amino acid transporter and converted to KYNA in the brain by astrocytes [59]. Thus, JM6 likely provides neuroprotection and antidepressant effects through increasing KYNA in the brain. The kynurenine pathway provides a connection between immune activation, serotonergic deficiency and NMDA receptor dysfunction that are involved in MDD. KMO shifts the metabolism of kynurenine toward the NMDA receptor agonist, quinolinic acid, increasing apoptosis and excitotoxic neurodegeneration. JM6 blocks KMO, shifting the metabolism of kynurenine toward the kynurenine-amino-transferase-3 pathway, which increases the formation of the NMDA receptor antagonist, KYNA, protecting against excitotoxicity produced by quinolinic acid [60]. This biochemical pathway for the mechanism of action of JM6 supports the hypothesis that JM6 is both neuroprotective and antidepressive. These properties of JM6, demonstrated by our results, support our hypothesis that gene and miRNA microarray expression profiles can provide signatures of mechanisms of action of novel compounds that can predict therapeutic properties. The intriguing findings of this pilot study merit further investigation and may improve future efforts to identify novel bioactive compounds with antidepressant and/or neuroprotective properties.

## Supporting information

S1 Table Pathways with significant overlap of genes differentially expressed in TBI+JM6/TBI and targeted by miRNA differentially expressed in opposite directions in TBI+JM6/TBI. (XLSX)

S1 File References in the caption for Fig 3C (Nos. 61–75) are included in the list, “Supplementary References for Fig 3C”. These references continue in the sequence established in the manuscript.
(DOC)
