# Supplementary material for: MicroRNA profiling identifies a novel compound with antidepressant properties
Source: PLoS One. 2019 Aug 23;14(8):e0221163. doi: 10.1371/journal.pone.0221163 (PMC6707633; doi:10.1371/journal.pone.0221163)
Supplement: S1 File — These references continue in the sequence established in the manuscript. (DOC) [file pone.0221163.s002.doc]

**Supporting Information**

Supplementary References for Figure 3C

61. Kahl KG, Georgi K, Bleich S, Muschler M, Hillemacher T, Hilfiker-Kleinert D, et al. Altered DNA methylation of glucose transporter 1 and glucose transporter 4 in patients with major depressive disorder. J Psychiatr Res. 2016;76:66-73. Epub 2016/02/27. doi: 10.1016/j.jpsychires.2016.02.002. PubMed PMID: 26919485.

62. Watkeys OJ, Kremerskothen K, Quide Y, Fullerton JM, Green MJ. Glucocorticoid receptor gene (NR3C1) DNA methylation in association with trauma, psychopathology, transcript expression, or genotypic variation: A systematic review. Neurosci Biobehav Rev. 2018;95:85-122. Epub 2018/09/04. doi: 10.1016/j.neubiorev.2018.08.017. PubMed PMID: 30176278.

63. Su M, Hong J, Zhao Y, Liu S, Xue X. MeCP2 controls hippocampal brain-derived neurotrophic factor expression via homeostatic interactions with microRNA132 in rats with depression. Mol Med Rep. 2015;12(4):5399-406. Epub 2015/08/05. doi: 10.3892/mmr.2015.4104. PubMed PMID: 26239616.

64. Jacobs JM, Traeger L, Eusebio J, Simon NM, Sequist LV, Greer JA, et al. Depression, inflammation, and epidermal growth factor receptor (EGFR) status in metastatic non-small cell lung cancer: A pilot study. J Psychosom Res. 2017;99:28-33. Epub 2017/07/18. doi: 10.1016/j.jpsychores.2017.05.009. PubMed PMID: 28712427.

65. Chiesa A, Crisafulli C, Porcelli S, Han C, Patkar AA, Lee SJ, et al. Influence of GRIA1, GRIA2 and GRIA4 polymorphisms on diagnosis and response to treatment in patients with major depressive disorder. Eur Arch Psychiatry Clin Neurosci. 2012;262(4):305-11. Epub 2011/11/08. doi: 10.1007/s00406-011-0270-y. PubMed PMID: 22057216.

66. Liu Q, Sun NN, Wu ZZ, Fan DH, Cao MQ. Chaihu-Shugan-San exerts an antidepressive effect by downregulating miR-124 and releasing inhibition of the MAPK14 and Gria3 signaling pathways. Neural Regen Res. 2018;13(5):837-45. Epub 2018/06/05. doi: 10.4103/1673-5374.232478. PubMed PMID: 29863014; PubMed Central PMCID: PMCPMC5998613.

67. Liu JJ, Sudic Hukic D, Forsell Y, Schalling M, Osby U, Lavebratt C. Depression-associated ARNTL and PER2 genetic variants in psychotic disorders. Chronobiol Int. 2015;32(4):579-84. Epub 2015/03/24. doi: 10.3109/07420528.2015.1012588. PubMed PMID: 25799324.

68. Landgraf D, Long JE, Welsh DK. Depression-like behaviour in mice is associated with disrupted circadian rhythms in nucleus accumbens and periaqueductal grey. Eur J Neurosci. 2016;43(10):1309-20. Epub 2015/09/29. doi: 10.1111/ejn.13085. PubMed PMID: 26414405.

69. Szopa A, Doboszewska U, Herbet M, Wosko S, Wyska E, Swiader K, et al. Chronic treatment with caffeine and its withdrawal modify the antidepressant-like activity of selective serotonin reuptake inhibitors in the forced swim and tail suspension tests in mice. Effects on Comt, Slc6a15 and Adora1 gene expression. Toxicol Appl Pharmacol. 2017;337:95-103. Epub 2017/11/07. doi: 10.1016/j.taap.2017.10.020. PubMed PMID: 29107002.

70. Dallaspezia S, Locatelli C, Lorenzi C, Pirovano A, Colombo C, Benedetti F. Sleep homeostatic pressure and PER3 VNTR gene polymorphism influence antidepressant response to sleep deprivation in bipolar depression. J Affect Disord. 2016;192:64-9. Epub 2015/12/29. doi: 10.1016/j.jad.2015.11.039. PubMed PMID: 26707349.

71. Xu YJ, Sheng H, Wu TW, Bao QY, Zheng Y, Zhang YM, et al. CRH/CRHR1 mediates prenatal synthetic glucocorticoid programming of depression-like behavior across 2 generations. FASEB J. 2018;32(8):4258-69. Epub 2018/03/16. doi: 10.1096/fj.201700948RR. PubMed PMID: 29543532.

72. Zarcone D, Corbetta S. Shared mechanisms of epilepsy, migraine and affective disorders. Neurol Sci. 2017;38(Suppl 1):73-6. Epub 2017/05/21. doi: 10.1007/s10072-017-2902-0. PubMed PMID: 28527083.

73. Carlberg L, Schosser A, Calati R, Serretti A, Massat I, Papageorgiou K, et al. Association study of CREB1 polymorphisms and suicidality in MDD: results from a European multicenter study on treatment resistant depression. Int J Neurosci. 2015;125(5):336-43. Epub 2014/06/24. doi: 10.3109/00207454.2014.936554. PubMed PMID: 24955721.

74. Antypa N, Souery D, Tomasini M, Albani D, Fusco F, Mendlewicz J, et al. Clinical and genetic factors associated with suicide in mood disorder patients. Eur Arch Psychiatry Clin Neurosci. 2016;266(2):181-93. Epub 2015/12/03. doi: 10.1007/s00406-015-0658-1. PubMed PMID: 26626456.

75. Zhang JC, Yao W, Hashimoto K. Brain-derived Neurotrophic Factor (BDNF)-TrkB Signaling in Inflammation-related Depression and Potential Therapeutic Targets. Curr Neuropharmacol. 2016;14(7):721-31. Epub 2016/01/21. PubMed PMID: 26786147; PubMed Central PMCID: PMCPMC5050398.
